# Educating the public health workforce: Issues and challenges

**DOI:** 10.1186/1743-8462-6-8

**Published:** 2009-04-09

**Authors:** Mary Louise Fleming, Elizabeth Parker, Trish Gould, Melinda Service

**Affiliations:** 1 School of Public Health, Queensland University of Technology, Brisbane, Queensland, Australia

## Abstract

**Background:**

In public health, as well as other health education contexts, there is increasing recognition of the transformation in public health practice and the necessity for educational providers to keep pace. Traditionally, public health education has been at the postgraduate level; however, over the past decade an upsurge in the growth of undergraduate public health degrees has taken place.

**Discussion:**

This article explores the impact of these changes on the traditional sphere of Master of Public Health programs, the range of competencies required at undergraduate and postgraduate levels, and the relevance of these changes to the public health workforce. It raises questions about the complexity of educational issues facing tertiary institutions and discusses the implications of these issues on undergraduate and postgraduate programs in public health.

**Conclusion:**

The planning and provisioning of education in public health must differentiate between the requirements of undergraduate and postgraduate students – while also addressing the changing needs of the health workforce. Within Australia, although significant research has been undertaken regarding the competencies required by postgraduate public health students, the approach is still somewhat piecemeal, and does not address undergraduate public health. This paper argues for a consistent approach to competencies that describe and differentiate entry-level and advanced practice.

## Introduction

The growth of undergraduate public health education in Australia has paralleled, but is not necessarily a consequence of, discussions about the requirements for a flexible health workforce to meet contemporary and future health challenges. Health workforce shortages and calls for renewal of the health workforce in Australia have been well-documented. The Australian Government Productivity Commission was appointed by the Council of Australian Governments (COAG in June 2004) to produce a comprehensive research report that outlined the breadth of the trends, issues and challenges in the health workforce in the next 10 years, including efficiencies and effectiveness and the need for innovation. Consultations and submissions were extensive and the Productivity Commission Health Workforce Study was released in January 2006 [1].

Subsequent to this report, "Health Workforce Australia" was established as a committee of the Australian Health Ministers Advisory Council (AHMAC) in late 2007. It claims that "the issues experienced at a national level include workforce shortages, maldistribution, managing changing models of care and maintaining a culture of effective governance and continuous improvement", and its objective is to ensure that our health system most effectively uses a skilled workforce to best support service delivery to all Australians [2].

Discussions about the need for a flexible public health workforce have been propelled not only by the above national developments but also by internal debates within the public health community about a set of competencies that would best meet the changing health profile of Australians. In addition, the evidence of effective public health investments to reduce the burden of premature mortality and morbidity has impacted on public health workforce requirements [3,4].

Furthermore, the new government is committed to a National Preventative Health Strategy, to be developed by a National Preventative Health Taskforce that will focus initially on the burden of chronic diseases, particularly the contribution of alcohol, tobacco and obesity [5]. With the spotlight on prevention within the policy agenda of the new government, discussion is warranted on the skills, competencies and attributes necessary for the public health workforce, as well as an analysis of current educational opportunities needed to fulfil the potential gaps in this workforce.

### The public health workforce: The changing nature of practice

Public health's scope of responsibility is broad and ever-increasing, such that public health practitioners require a variety of skills; many, however, have had insufficient education or training regarding how to address increasingly diverse and emergent public health challenges [6,7]. The skills and proficiencies required include a basic insight as to what public health is, what it does, and how it accomplishes its aims. In addition, core competencies that impart knowledge and expertise regarding all spheres of public health practice are required; as is specialised "know-how", which provides the precise knowledge and expertise necessary for specific programs or functions. Although there are a variety of views as to what these specific competencies are or should be, there has been significant research undertaken by a range of organisations to facilitate the attainment of consensus on the essential skills needed for public health practitioners.

The roles and functions of the public health workforce are diverse in nature, as they are influenced by the contexts within which they are set [8]. This varied make-up of the workforce offers both possibilities and challenges for public health training [9]. The challenges result from deficiencies in employee competencies; that is, those necessary for existing duties or those required to deal with evolving problems [9]. The capacity to pinpoint student destinations and utilise relevant approaches to public health workforce development is vital [9,10].

Public health practitioners can come from science, humanities and arts; and this multidisciplinary composition is one of its strengths because the protection of populations requires a comprehension of, for example, the environmental, social and political foundations of health. This is also a limitation, as there are insufficient shared core competencies across different fields [11]. Moreover, the tasks are rarely standardised, entailing distinct combinations of expertise to isolate and assess the origins of public health challenges, provide useful approaches to these, and the capacity to judge their "impact and effectiveness" [8].

Public health education ought to be provided to the entire public health workforce; from the undergraduate level, to in-service training for established public health workers, through to postgraduate level. Furthermore, fundamental public health concepts and skills should be taught to all workers employed in health-related positions, as well as to people working in areas traditionally not seen as being part of the health sector [12] such as town planning and transport. US working groups (Association for Prevention, Teaching and Research, Association of Schools of Public Health, Council of Colleges of Arts and Sciences) go even further, recommending that introductory courses in public health and epidemiology should be available to all undergraduates, not just those in public health [13,14].

The increasing need for practitioners with recognised qualifications in public health has generated the growth in education and training programs. Progress in public health disciplines and an increased range of employment opportunities has led to curricula that meet a more diverse range of needs, as the challenges and problems of public health become progressively more complicated [15]. The quality of public health education is gaining in importance due to the increasingly diverse activities that professionals may have to undertake and the expansion in required skills [16].

A degree in public health serves to enhance graduate skills and the application of those skills, fulfil requirements for promotion and facilitate movement within the health sector. Traditionally, the principal employers of these graduates were the government sector – at national, state and local levels; and the non-government sector, such as the Heart Foundation, the Cancer Fund and Diabetes Australia. However, prospects for those with qualifications in public health are increasingly diverse, with a wide range of enterprises employing public health practitioners. Current public health practice has expanded to embrace a range of different environments; for example, voluntary organisations, diverse commercial/industrial sites, community-based groups and health care services.

Internationally, authors have discussed the need to increase expertise in the public health workforce if practitioners are to meet the diverse requirements of their roles in protecting and advancing health [16]. Workforce capacity reviews indicate that there are shortages of people with the necessary skills and knowledge [16,17]. However, developing the workforce is a complex task because of the numerous responsibilities that come under the aegis of public health, and the wide-ranging skills that are considered essential [16]. It is timely to consider how public health education meets the challenges of a diverse and increasingly complex workforce.

### The impact of competencies on curriculum development

There are a number of examples, both nationally and internationally, of activity that has occurred to establish core areas of activity or competencies in public health. In 1997, the World Health Organization performed a Delphi study that delineated 37 vital public health functions, and established that it was possible to achieve wide-reaching agreement on these core areas [18].

In Europe, the Association of Schools of Public Health (ASPHER) in the European Region met in Denmark in April 2008 to build on work that had begun within the European Union where projects examined public health courses, competencies and accreditation: "An MPH accreditation document was developed in 2002 by ASPHER and a set of standards and procedures has now led to a set of Accreditation Standards (EU Accreditation of European Public Health Education; MPH Programme Standards)" [19]. This activity was driven in part by the Bologna Declaration. Signed in 1999 by European Union (EU) higher education ministers, the Bologna Process aims to create a European Higher Education Area (EHEA) based on international cooperation and academic exchange that is attractive to European students and staff, as well as to students and staff from other parts of the world [20].

In the United States, core competencies for public health curriculum have been a well-established component of the academic and practice landscape. The Council on Linkages Between Academia and Public Health Practice developed the "Core Competencies for Public Health Professionals" to help strengthen public health workforce development [21]. This builds on 10 years' work on this subject by the Council and numerous other organisations and individuals in public health academic and practice settings. The list has been compared with the "Essential Public Health Services" [22] to ensure that the competencies help build the skills necessary for service delivery.

A consensus set of core competencies for guiding public health workforce development has been achieved in the US. These competencies include analytic/assessment, policy development/program planning, communication, cultural competency, community dimensions of practice, basic public health sciences, financial planning and management and leadership and systems thinking.

In the United Kingdom, the Faculty of Public Health identifies the curriculum areas outlining the competencies or learning outcomes that trainees in public health need to attain in order to complete their training. These nine key areas relate to the three domains of public health practice (health protection, health improvement and service quality). These key areas include "surveillance and assessment of the population's health and wellbeing; assessing the evidence of effectiveness of health and healthcare interventions, programmes and services; policy and strategy development and implementation; strategic leadership and collaborative working for health; health intelligence and academic public health" [23].

In Australia, a number of projects have examined the development of core competencies. For example, health promotion competencies were developed in the early 1990s in Western Australia. More recently, in 2006, an extensive consultation process resulted in the development of competencies for health promotion practice in Australia [24].

Another project, managed by the National Public Health Partnership Group, surveyed Australian public health experts regarding their views on defining public health functions. The aim was to help identify essential public health functions and develop Australian public health capacity [25]. PHERP competencies are intended for PHERP-funded universities offering generalist Masters in Public Health. Five broad categories recognise the major themes for public health education and 19 public health units of competency are subsumed under these five broad categories. The categories are: Health Monitoring and Surveillance; Disease Prevention and Control; Health Protection; Health Promotion; and Health Policy, Planning and Management [26]. This work is yet to be agreed upon by PHERP-funded institutions and then brought to a conclusion.

### The public health education response

The majority of public health courses have been at the postgraduate level both in Australia [18] and internationally [27,28]. Accordingly, admission into public health education has generally been open to people with a first degree in a range of professional or academic fields and/or relevant work experience [18,27].

In the past decade in Australia the number of universities offering dedicated undergraduate public health programs has expanded [18]. Mor et al. [29] suggested that increasing attention to public health and health promotion in contemporary society is driving an interest in undergraduate public health curricula in the United States. In part, this has also occurred in tertiary institutions in Australia. However, there is limited discourse in the Australian context around the implications of these developments – some of which are introduced below and covered in detail later in the paper:

• Does the expansion in undergraduate public health education impact on the nature and scope of the postgraduate public health curriculum?

• Are the curricula influenced by competencies?

• Do curricula complement each other or are there clear differences?

• If there are differences are they a matter of content, level or both?

• What are the expectations vis-à-vis undergraduate programs, compared with graduate level programs that have had as their focus the development of public health leadership skills?

• Are there any differences in the employment prospects for students who complete undergraduate degrees compared with those who complete postgraduate degrees?

• Are there any differences in graduate capabilities?

The research pertaining to undergraduate public health education is limited compared to that of postgraduate public health education, nursing-related public health education and public health training for medical students. There is abundant research and commentary with regard to competencies for Master of Public Health education and training, and some consideration of specific subjects or competencies that should be taught to undergraduates; for example, "cultural competence" [30], and "problem-based learning" skills [31]. However, there is a dearth of literature regarding the differences between undergraduate and postgraduate public health education. Are there implications for postgraduate education for those students who have completed a public health undergraduate degree?

"Undergraduate public health education" in this commentary refers to distinct public health education, not other health courses that incorporate public health subjects; for example, nursing. Postgraduate education usually refers to the Master of Public Health. Other postgraduate education in health sciences or health management, for example, includes public health units but does not involve a comprehensive range of specific units in the discipline area.

### Developments in undergraduate public health education: The international scene

The information presented below is a snapshot of a range of tertiary institutions in a number of countries that offer undergraduate education in public health.

In the US, undergraduate students' interest in public health is stimulated by a range of global issues including the rise in both pandemic and chronic diseases [11,32], risks to biosecurity [28,32], technological advances requiring new expertise, ecological damage and catastrophes, demographic changes [32], and the high proportion of public health professionals with limited formal public health training [11,28].

Undergraduate public health education can be seen as a natural outgrowth of the rise in interdisciplinary undergraduate education – with insights regarding influences on public health having roots in a variety of subjects, including economics, psychology, anthropology and biology [28]. Conversely, the population approach of public health, and the generic expertise and knowledge gained, provide a good basis for a variety of professions, and postgraduate study in a range of disciplines, including social work, law and health policy [28].

For example, there are over 40 undergraduate public health programs in the US [29] that demonstrate a wide and varied curriculum even though there is a new accreditation process for undergraduate programs (managed by the Council on Education for Public Health [CEPH]) should a university choose this option. This new accreditation process has the "potential to radically change the scope of undergraduate public health education" [14]. One of the CEPH's requirements for accreditation is that the university must be focussed on "training students for entry-level public health positions" [14].

By contrast, a survey of Canadian universities retrieved only one undergraduate public health program. None of the five South African universities scanned (Universities of Cape Town, Johannesburg, Pretoria, South Africa and the Witwatersrand) offered undergraduate public health programs; although, the following three universities provided MPH or MA (Public Health) programs – Cape Town, South Africa, the Witwatersrand.

The Asia Pacific Academic Consortium of Public Health (APACPH), representing public health schools in over 20 countries in the region, has a working party examining undergraduate public health curricula across its member institutions to consolidate commonalities, gaps and competencies in order to integrate public health education with workforce needs to address public health priorities [33].

### Developments in public health education: The Australian scene

In Australia, undergraduate public health education has been available at some universities since the 1990s. For example, Adelaide University graduated its first Bachelor of Health Science (Public Health) cohort in 1992 [18].

Nine Australian universities offer public health degrees or health degrees with a significant public health component. Six of these universities have the option of combining the public health degree with a degree in another faculty or school; for example, Nursing, Human Movements, and Creative Industries.

Many commentators agree that there ought to be shared foundation subjects for all students of public health, and there is some consensus regarding the core of topics that are essential; however, a comprehensive approach to competencies at the undergraduate level is lacking in Australia compared with the United States [29]. In the Australian context, because there are neither set competencies nor an accreditation body, there are degrees of freedom around what is taught; although many of these programs would advocate that students could be employed as public health workers on completion of the course.

Undergraduate public health courses usually attract school-leavers, undergraduates with limited work experience, or undergraduates from a range of disciplines not necessarily health-related (e.g. planning, architecture) with an interest in health. Programs offer a broad introduction to public health concepts and themes, research techniques, informatics and communication techniques.

On the other hand, postgraduate public health courses generally attract graduates from a range of disciplines, graduates already in the workforce in health-related occupations, such as health services management and medicine, and non-graduates in health-related fields looking to upgrade their qualifications and skills. These programs prepare students for middle and upper-level management by providing more advanced skills and knowledge in research methodology, administration, leadership, human resources and advanced global health policy and politics, than do undergraduate courses.

Postgraduate public health programs provide a preparation for specific careers such as health manager, policy adviser, environmental health officer, health promotion officer, or in teaching and research. They also provide opportunity to specialise [34] and for advanced work-based projects. In addition, there are opportunities for medical doctors, or other health professionals, who want more specific public health skills and knowledge (e.g. epidemiology, research).

### Emerging tensions: Differences and similarities in public health education in Australia

Throughout Australia, the rise in students participating in undergraduate public health education raises the issue of the comparative value of undergraduate versus postgraduate programs in public health. The role of undergraduate public health education in professional development necessitates further exploration [18]. What happens when students with undergraduate public health qualifications want to enrol in a Master of Public Health degree? Does this mean that a university might offer two MPH programs? The first being one that includes advanced statistics and epidemiology units, and policy and leadership studies for students who have an undergraduate public health degree. The second program, designed to meet the needs of a range of health and other professionals, might be similar to what is now offered as a generic MBA program.

How might we describe the workplace opportunities and the skills of the entry level practitioner who has completed an undergraduate degree? They might begin their working life as a project officer, while the student who completes a postgraduate degree, with more life and work experience, may begin their public health working life at the policy or middle management level.

If an undergraduate public health qualification is considered equivalent to a Master of Public Health qualification, what are the implications? It may be that professionals from a range of disciplines (e.g. medicine, nursing) will be discouraged from undertaking further education in public health. Furthermore, it is inevitable that there will be duplication in the competencies taught across both levels, thus raising the issue of whether a student with an undergraduate public health degree will benefit from undertaking a postgraduate degree in public health. For example, epidemiology and biostatistics are core knowledge areas for public health. The question then arises as to whether these topics would have the same focus for both the MPH student and the undergraduate if both are considered "beginning practitioners".

An analysis of undergraduate public health programs around Australia indicate that they are designed for entry-level practitioners. Typically these programs provide: an overview of the scope of public health responsibilities; the foundation knowledge for specialist fields at postgraduate level; an overview of a range of disciplines that contribute to health protection and promotion; an opportunity to enter the program straight from school without prior work experience; a coverage of generic skills (associated with a less specialised tertiary-level education) rather than the skills more specific to public health [18].

One of the emerging issues for schools of public health with an MPH program is the expansion of undergraduate teaching in public health. For those schools where undergraduate public health programs have emerged, and where they also teach postgraduate public health, the similarities or differences between the curricula present real problems. A number of questions arise: What do we consider is entry level for the public health workforce? How different are practitioners with an undergraduate as opposed to a postgraduate degree in public health? Should undergraduate degrees be more generic qualifications while postgraduate degrees represent advanced-level competencies and how does this influence employment and practice?

It is important that a distinction is made between the educational requirements of undergraduate and postgraduate students [18]. Specifically, approaches are needed that improve the relevance of undergraduate public health education to workforce needs [18]. Furthermore, undergraduate-level public health programs should not merely replicate postgraduate-level programs [18,28]. Postgraduate public health programs need more education in the areas of policy leadership; emerging issues, such as Avian influenza; strategies and activities that promote awareness and protect the population from developing, for example, chronic diseases; and preparedness training for emergencies. In their survey of graduates from the Adelaide University undergraduate public health program, Houghton determined that the generic competencies achieved through their undergraduate public health education were valued by the students over the more public health-specific skills and knowledge [18].

Schlaff and Chang [7] pointed out the difficulty in attaining both the depth and breadth in public health education at the postgraduate level recommended by the Institute of Medicine report [34]. This challenge could be resolved by emphasising breadth of knowledge at the undergraduate level, while focusing on depth, depending on the person's specific requirements, at the postgraduate level.

### Implications

Without an agreed definition of the fundamental responsibilities of public health practice and the consequent knowledge and proficiencies required of public health professionals, it is difficult to define an appropriate curriculum.

A number of issues arise for curriculum designers. First, thoughtful mapping of the curricula and *levels *of competencies is needed for those students wishing to articulate from an undergraduate public health degree into a postgraduate degree. As discussed earlier in this commentary, this issue is particularly important for examining the core sets of knowledge and skills of epidemiology and biostatistics. Those with undergraduate degrees in public health would be well-placed to tackle advanced epidemiology and a breadth of advanced research studies in qualitative research, social research, health economics, strategic leadership, and change management; as well as retaining a discrete focus on health monitoring and surveillance, health protection, disease prevention and control, health policy planning and management and health promotion. There should be an expectation that students graduate with high levels of analytical and conceptual skills.

Second, health care sector engagement is pivotal for advancing curricula, especially in anticipating future needs, and the skills and attributes required of graduates. The use of industry liaison groups, including international industry partners and course advisory groups to link all graduates to employment may be an important strategy for increasing collaboration between the workplace and the university sector.

Third, workforce mobility is an important consideration for graduates of public health programs. For example, a student of an undergraduate public health program may choose not to advance their public health skills, but rather engage in education in management and policy. To date, limited information exists about the employment profiles of students who complete undergraduate programs in Australia to enable tracking of their employment over the past five years to gain further understanding of the choices undergraduate students make.

The links between workforce readiness and university education cannot be underestimated, as health employers develop their own competencies. For example, some state health departments have developed entry-level and advanced competencies for public health workers, without reference to university curricula. In addition, a range of other groups have developed benchmarks for graduates' employability skills and attributes. For example, the "Employability Skills for the Future" developed by the Australian Chamber of Commerce and Industry and the Business Council of Australia; Graduate Employability Skills as prepared for the Business, Industry and Higher Education Collaboration Council (2007); and the Graduate Attributes as described by all universities.

## Conclusion

The issues addressed above attempt to answer many of the questions that were raised earlier in this commentary. With the emergence of a number of undergraduate public health programs in Australia one of the challenges is to ensure that students are educated to meet the needs of a flexible and dynamic public health workforce. Employers, professional associations and universities need to work together to ensure that needs are met for entry-level practice with the option of developing more advanced knowledge and skills in areas such as epidemiology and statistics and policy and management at a postgraduate level. Also of importance is the role of the employer in continuing professional development in the workplace, in particular for those public health workers who are entry-level practitioners. Universities and employers need to continue to work together to ensure that the field is well-represented by competent and capable public health practitioners who can work in an ever-changing environment to advance the health of the population.

## Competing interests

The authors declare that they have no competing interests.

## Authors' contributions

TG completed the initial draft of the manuscript. All authors had significant roles in conceiving the manuscript and participated in revising it critically for intellectual content.
